# 

**Published:** 1880-05

**Authors:** 


### CONTENTS:

ARTICLE I.—New Methods of Mounting Artificial Teeth. Continued............ 1

ARTICLE II.—Researches on Hearing through the Medium of the Teeth and Cranial Bones. By Chas. Hermon Thomas, M. D............ 9

ARTICLE III.—Neuralgia and its Treatment. By Truman W. Brophy, D. D. S........17

ARTICLE IV.—Death of the Dental Pulp by External Violence. By Dr. G. Dop. . ...31

ARTICLE V.—Extraction of First Molars. By Dr. A. W. Harlan..........38

### EDITORIAL, ETC.

Mechanical Dentistry Items..........41

A Valuable New Compound Metal..........43

### MONTHLY SUMMARY.

Proof of Death..........44

Compressed Nitrous Oxide as an Anæsthetic..........44

A New Disinfectant..........45

Chloroform in Diseases of the Heart..........45

Cement for Mending Hard Rubber..........45

Treatment of Salivary Fistula..........46

Source of Muscular Power..........46

Precautions in Anæsthesia..........46

Mesmeric Anæsthesia..........47

Manufacture of Celluloid..........47

To Check Dental Hemorrhage..........47

The Effects of Smoking upon the Teeth..........47

Asbestos Powder as a Cement..........48

An Anti-Scorbutic..........48

The New Anæsthetics..........48

Rubber Cement..........48

### OBITUARY.

Drs. S. P. Cutter and J. C. Harris..........48

